# Reporting-source differences in associations between student-perceived relationships with teachers and social–emotional skills: evidence from the Suzhou SSES 2019 sample

**DOI:** 10.3389/fpsyg.2026.1949088

**Published:** 2026-10-14

**Authors:** Runiu Zhu, Bohan Wang, Xiaofei Chen, Xiaoyi Kong

**Affiliations:** 1 Department of Neurology (Sleep Disorders), The Fourth Affiliated Hospital of Anhui Medical University, Hefei, Anhui, China; 2 School of Educational Science, Anqing Normal University, Anqing, China

**Keywords:** educational psychology, multi-informant assessment, OECD survey on social and emotional skills, social–emotional skills, teacher–student relationships

## Abstract

**Introduction:**

Students’ perceptions of their relationships with teachers may be associated with social-emotional functioning, but the magnitude of this association may depend on who reports the outcomes. This study examined whether student-perceived relationships with teachers were associated with five domains of social-emotional skills reported by students, parents, and teachers, and whether association magnitudes differed across reporting sources.

**Methods:**

Data were drawn from 7,246 students in 151 school-by-cohort units in the Suzhou sample of the 2019 OECD Survey on Social and Emotional Skills. Survey-weighted fixed-effects models estimated within-unit associations, matched stacked models compared slopes across informants, and school-by-cohort-grouped cross-validation assessed incremental prediction.

**Results:**

All 15 informant-by-domain associations were positive and remained significant after false-discovery-rate correction. In matched samples, student-report slopes ranged from 0.234 to 0.256, compared with 0.083 to 0.109 for parent reports and 0.080 to 0.106 for teacher reports. All student-versus-adult contrasts remained significant after correction, whereas none of the parent-versus-teacher contrasts did. The same pattern was observed in 44 of 45 skill-level tests. Adding the relationship index improved out-of-fold weighted R² in all 30 prediction comparisons, although absolute predictive performance was weak, particularly for adult-reported outcomes.

**Discussion:**

Student-perceived relationships with teachers showed cross-informant relevance, while the strongest observed associations occurred when both the relationship measure and the social-emotional outcome shared the student reporter. These findings suggest that reporting source should be treated as substantive information in educational assessment and that relationship ratings should be used to contextualize, rather than replace, multi-informant evidence.

## 1 Introduction

Social–emotional skills shape how students pursue goals, regulate emotions, engage with others, approach unfamiliar ideas, and collaborate. The OECD Survey on Social and Emotional Skills (SSES) organizes 15 social–emotional skills into five broad domains: task performance, comprising responsibility, self-control, and persistence; emotional regulation, comprising stress resistance, emotional control, and optimism; engaging with others, comprising energy, assertiveness, and sociability; open-mindedness, comprising curiosity, creativity, and tolerance; and collaboration, comprising empathy, co-operation, and trust (Kankaraš and Suárez-Álvarez, 2019; OECD, 2021a). Across SSES sites, the skills are associated with achievement, belonging, wellbeing, health, and other educational outcomes, although the most informative skills and the magnitude of their associations vary across outcomes, cultures, ages, and reporting sources (Altiner Sert and Arıkan, 2025; Guo et al., 2023; Huang et al., 2024; Meng et al., 2025; Niu, 2024, 2025). For educational psychologists and other professionals who interpret school-based assessment, this heterogeneity matters because decisions often integrate social–emotional evidence obtained from different people and settings.

Relationships with teachers are a central component of those environments. Several complementary theoretical perspectives explain why teacher–student relationships should be associated with students’ social–emotional functioning. Developmental-systems accounts conceptualize teacher–student relationships and student adjustment as reciprocally embedded in ongoing classroom interactions, rather than as a simple one-directional process (Sabol and Pianta, 2012). From an attachment-informed perspective, a sensitive and supportive teacher may function as an *ad hoc* source of security in the school context, providing a “secure base” that facilitates exploration and coping. However, teacher–student relationships should not generally be equated with attachment bonds (Verschueren and Koomen, 2012). Self-determination theory provides a complementary motivational account: perceived teacher care, involvement, and responsiveness may support students’ need for relatedness and, together with support for competence and autonomy, foster engagement and self-regulation (Niemiec and Ryan, 2009). The Prosocial Classroom model further proposes that supportive teacher–student relationships form part of a classroom environment that can promote students’ social and emotional development (Jennings and Greenberg, 2009). Together, these perspectives suggest several non-exclusive pathways through which perceived relationship quality may be associated with social–emotional skills, including greater emotional security, stronger engagement and receptiveness to feedback, and more opportunities to observe, practice, and co-regulate emotional and interpersonal behavior. These mechanisms are theoretical possibilities rather than processes directly tested in the present cross-sectional study.

Consistent with these theoretical perspectives, longitudinal and meta-analytic evidence links relationship quality with engagement, achievement, social competence, behavior, and wellbeing (Emslander et al., 2025; Hamre and Pianta, 2001; Magro et al., 2024; Roorda et al., 2011, 2017). Recent research has also linked teacher–student relationships to adolescents’ distress and mindfulness and documented longitudinal patterns linking relationship quality to social–emotional development (Mills-Webb and Hennessey, 2025; Pastore et al., 2025). Other studies connect culturally responsive relationships, teacher care, support, motivational climate, and perceived relationships with social–emotional competence or mental health (Boat et al., 2025; Ma et al., 2026; Tennant et al., 2015; Zhang et al., 2026; Zhao et al., 2026; Zhou et al., 2025). The reverse pathway is also plausible: students who persist, regulate emotions, or communicate effectively may elicit different teacher responses and evaluate classroom interactions more positively. Reciprocal longitudinal findings reinforce this possibility (Magro et al., 2026; Yang et al., 2023). A cross-sectional association should therefore be treated as a relational correlate, not as an identified developmental effect.

The Chinese educational context also provides a meaningful setting for examining these associations. Teacher–student relationships are embedded in culturally shaped expectations about interpersonal roles and classroom interaction. Cross-cultural research shows that Chinese and Western students and teachers may differ in perceived relationship closeness, conflict, and cross-informant agreement, suggesting that findings on teacher–student relationships should not automatically be assumed to transfer unchanged across educational cultures (Chen et al., 2019). Suzhou is particularly relevant within the present dataset because it was the Chinese site participating in SSES 2019. The OECD’s Suzhou site report also documents a strong local emphasis on social–emotional skills and the incorporation of social–emotional learning into primary and secondary education within broader national initiatives (OECD, 2021c). These features make Suzhou a substantively relevant context for examining links between perceived relationships with teachers and social–emotional skills, but they do not make the city representative of China as a whole. Accordingly, the present study treats Suzhou as a context-specific case and does not assume that the observed association magnitudes will generalize unchanged to other Chinese cities or educational systems.

This interpretive problem is sharpened by the reporting source. In SSES 2019, students rated how often most teachers treated them fairly, got along with them, and showed interest in their wellbeing (OECD, 2021a,b). The resulting indicator captures students’ general perceptions; it is neither direct observation of teacher behavior nor a dyadic measure tied to one teacher. Student perceptions remain educationally meaningful, but associations with student-reported skills may also reflect shared response styles, general evaluative tendencies, or semantic overlap (Podsakoff et al., 2003; Primi et al., 2019). Reviews further show that student-report relationship instruments vary substantially in construct coverage and psychometric support (Phillippo et al., 2017). Recent classroom research similarly found that relationship-mediated conclusions differed between students’ self-reported social integration and peer nominations, illustrating the value of distinguishing methods and informants (Rauterkus et al., 2025).

A multitrait-multimethod perspective treats this source variation as substantive information rather than assuming that one informant is a gold standard (Campbell and Fiske, 1959). Students can report internal feelings and experiences across teachers; parents observe behavior primarily outside school; and teachers observe behavior under classroom demands. Cross-informant agreement in child and adolescent assessment is usually low to moderate, and discrepancies can reflect context as well as error (De Los Reyes et al., 2015; De Los Reyes and Epkins, 2023; Renk and Phares, 2004). Studies of social–emotional competence similarly report patterned disagreement among students, parents, teachers, and observers (Asadi, 2026; Huber et al., 2019; Humphries et al., 2012; Javo et al., 2009; Mudarra et al., 2022; Ren and Fan, 2021). SSES evidence also indicates that conclusions change when different informants report skills (Guo et al., 2023). An association with students’ perceived relationships may thus persist across sources while remaining larger when predictor and outcome share the student reporter. This is a practical assessment question: informant discrepancies can guide context-focused inquiry by educators and educational psychologists, whereas treating ratings as interchangeable can obscure where and for whom a difficulty is observed.

These perspectives lead to two primary expectations. First, if supportive teacher–student relationships are linked with students’ emotional security, engagement, self-regulation, and opportunities for interpersonal learning, positive associations should not be confined to a single social–emotional domain or to students’ own ratings. Second, because the predictor is itself a student-perceived relationship measure, associations should be strongest for student-reported outcomes, which share the student’s perspective and response context. In contrast, smaller positive associations with parent- and teacher-reported outcomes would indicate that the pattern is not wholly confined to shared student reporting. We did not specify *a priori* rank-order hypotheses among the five domains or 15 individual skills because the proposed relational mechanisms are broad and non-exclusive.

The broader multi-informant literature has already shown that student, parent, and teacher reports may differ systematically and meaningfully, including in assessments of social–emotional functioning (De Los Reyes et al., 2015; De Los Reyes and Epkins, 2023; Mudarra et al., 2022). Existing SSES studies have linked relationships with teachers, feedback, teacher social–emotional training, sports participation, school climate, achievement, wellbeing, health, and bullying to social–emotional skills using conventional models or machine learning (Cui et al., 2026; Gao et al., 2024; Huang et al., 2024; Li and Qian, 2025; Lv et al., 2026; Meng et al., 2025; Wang and Qian, 2024; Yan and Lu, 2026). Among these studies, Huang et al. (2024) is particularly relevant because it also analyzed the SSES 2019 Suzhou survey setting and reported positive associations between teacher relationships and all 15 student-reported social–emotional skills. The student-report component of the present analysis should therefore be viewed as partly corroborating this prior Suzhou finding rather than as establishing that broad association *de novo*. This study addresses a more specific gap. The SSES studies reviewed above have not examined, within the same students, whether the association between a common student-perceived relationship-with-teachers measure and social–emotional outcomes differs in magnitude across student-, parent-, and teacher-reported outcomes. Thus, the present study focuses on matched, within-sample comparisons of association strength across reporting sources rather than on cross-informant agreement alone.

Within the SSES framework, the 15 individual social–emotional skills are organized into the five broad domains introduced above, with three component skills assigned to each domain (Kankaraš and Suárez-Álvarez, 2019; OECD, 2021a). In this study, we used the five domain-level outcomes as the primary summaries to provide a parsimonious basis for comparing associations across reporting sources. We then examined all 15 individual skills to assess whether the domain-level pattern was broadly distributed across component skills rather than driven by only a small subset. This distinction is important because previous network and validation studies suggest that the empirical organization of SSES skills may vary across cities and cultural contexts (Cheung and Ng, 2024; Huo and Ning, 2025; Lee and Junus, 2024; Wang and Ning, 2026). Accordingly, we treated the skill-level analyses as a breadth check rather than an attempt to revalidate the SSES five-domain structure.

Prediction provides a separate, secondary test of whether the relationship indicator contributes to out-of-sample prediction across held-out school-by-cohort units within the present Suzhou sample. Explanatory association and out-of-sample prediction are distinct properties (Shmueli, 2010; Yarkoni and Westfall, 2017). Random student splits can produce overly optimistic out-of-sample performance estimates when students from the same clustered setting appear in both training and test data; validation should instead respect the intended grouping structure (Roberts et al., 2017). Similarly, Shapley additive explanations (SHAP) values describe how a fitted model uses a predictor but do not identify causal importance or intervention leverage (Lundberg et al., 2020). Accordingly, grouped prediction is used here as a secondary internal-validation analysis rather than as evidence of external generalizability.

Taken together, the literature reviewed above shows that teacher–student relationships are associated with social–emotional outcomes, including student-reported SSES skills (Huang et al., 2024), while broader multi-informant research indicates that student, parent, and teacher ratings can differ systematically (De Los Reyes et al., 2015; De Los Reyes and Epkins, 2023; Mudarra et al., 2022). What remains unclear is whether, within the same students, the magnitude of the association between a common student-perceived relationship-with-teachers measure and social–emotional outcomes differs systematically across student, parent, and teacher reports. To address this gap, this study analyzed 7,246 students in 151 school-by-cohort units from the SSES 2019 Suzhou public-use data and compared source-specific associations in matched samples using the same relationship predictor and parallel social–emotional outcomes. Domain-level comparisons constituted the primary analysis, with skill-level analyses used to examine the breadth of the pattern and grouped prediction treated as secondary evidence (Figure 1). Two research questions organized the primary analysis.

**Figure 1:**
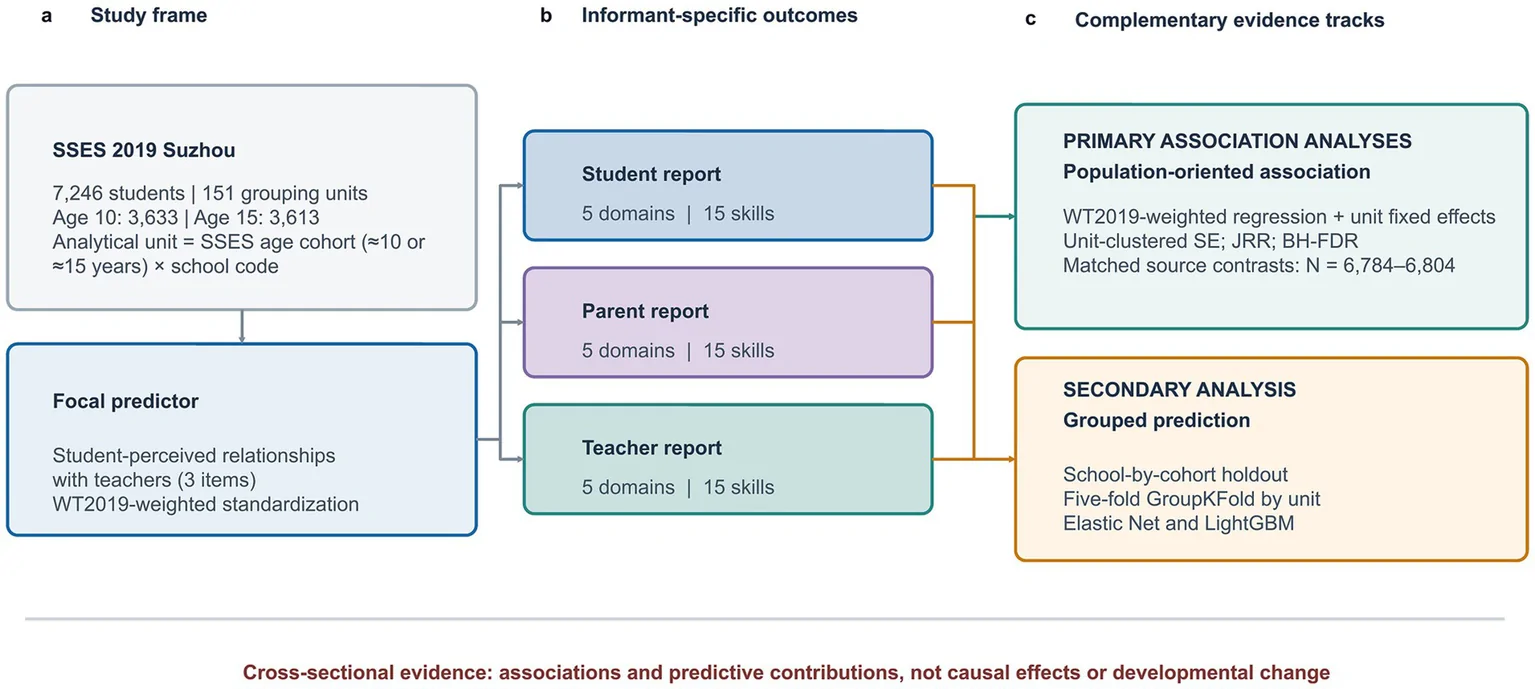
Study design and evidence hierarchy. The analytical frame comprised 7,246 students in 151 school-by-cohort units identifiable in the public-use data. Here, “cohort” refers to the two cross-sectional SSES age cohorts, approximately 10 and 15 years old; the analytical grouping unit was defined as SSES age cohort × school code. The primary analysis compared five domains and 15 skill outcomes reported by students, parents, and teachers using survey-weighted school-by-cohort fixed-effects models. Grouped prediction was a secondary analysis. All analyses were cross-sectional. SSES, Survey on Social and Emotional Skills; WT2019, the final student sampling weight provided in the SSES 2019 public-use data. “WT2019-weighted standardization” indicates that a variable was standardized using its WT2019-weighted mean and population standard deviation. SE, standard error; JRR, jackknife repeated replication; BH-FDR, Benjamini–Hochberg false-discovery-rate correction.

RQ1: Are student-perceived relationships with teachers positively associated with social–emotional skills reported by students, parents, and teachers? RQ2: Do the magnitudes of these associations differ across reporting sources in matched samples? Consistent with the theoretical account above, we expected positive associations across reporting sources, but stronger observed associations for student-reported outcomes because the predictor captures students’ own perceptions of their relationships with teachers and therefore shares the same perspective and response context with student-reported social–emotional outcomes. Skill-level models tested the breadth of the domain pattern. Moderation, functional-form, and school-by-cohort-grouped prediction analyses were treated as exploratory or secondary evidence. These latter analyses probed robustness, heterogeneity, functional form, and out-of-sample information rather than testing additional theory-derived hypotheses. Although the numerical estimates are context-specific, the comparison addresses a broader educational psychology problem: how reporting source changes the interpretation of relational and social–emotional assessment data.

## 2 Materials and methods

### 2.1 Study design and data source

This study was a secondary analysis of public-use data from the 2019 OECD SSES (OECD, 2021c). SSES assessed two cross-sectional age cohorts, approximately 10 and 15 years old, and collected information from students, parents, teachers, and school principals. Within each site and cohort, a two-stage stratified design sampled schools with probability proportional to size and then sampled students within schools (OECD, 2021a,b). Suzhou was the Chinese participating site in SSES 2019, and this analysis treats it as a city-specific case rather than a nationally representative Chinese sample (OECD, 2021c). We restricted all files to Suzhou (SiteID = “11”). The approximately 10- and 15-year-old groups were sampled as two distinct cross-sectional age cohorts in the same SSES 2019 survey round; they comprised different students rather than repeated observations of the same individuals. Accordingly, comparisons between the age cohorts cannot be interpreted as longitudinal change or individual developmental trajectories. We linked the student, parent, teacher indirect-assessment, teacher-questionnaire, and principal files. Student–parent and student–teacher assessment records were linked by the standardized student identifier; teacher-questionnaire data were linked by cohort, school, and teacher identifiers; and principal data were linked by site, cohort, and school. Because the technical documentation describes respondent identifiers as cohort-specific, we defined the finest defensible grouping unit in the public data as CohortID × SchID. Repeated numeric school codes across cohorts were not assumed to denote the same physical school. Key uniqueness and linkage cardinality were checked before and after every join, and direct student identifiers were removed from the analytical file.

### 2.2 Participants and analytical samples

The Suzhou student file contained 7,246 students in 151 school-by-cohort units: 3,633 students in 76 age-10 units and 3,613 students in 75 age-15 units. The numeric school field contained 122 codes, 29 repeated across cohorts; 122 is therefore not reported as a verified physical-school count. Parent records were matched to 7,080 students and teacher indirect assessments to 7,032. The official relationship-with-teachers index was available for 7,229 students. Complete-case domain samples included 7,225 students for student reports, 7,054–7,056 for parent reports, and 6,929–6,948 for teacher reports. Domain-specific samples containing all three informants included 6,784–6,804 students across all 151 units. For descriptive characterization of the informant samples, parent respondent identity was obtained from PAQM00201, teacher sex from TCQM00101, and teacher subject areas from the 12 multiple-response items TCQM01201–TCQM01212 (OECD, 2021b,c). Student-centered descriptive estimates used WT2019, parent respondent percentages used the parent final weight WT2019_PA, and unique-teacher percentages used the teacher final weight WT2019_TC. Missing and non-response codes were excluded from valid-percentage denominators, and teacher subject-area percentages were calculated separately because teachers could report more than one subject area.

### 2.3 Measures

#### 2.3.1 Student-perceived relationships with teachers

The focal predictor was the OECD-derived student index st_relteach, referring to the preceding 12 months. Three four-category items asked how often most teachers treated the student fairly, got along well with the student, and were interested in the student’s wellbeing. Responses ranged from 1 (never or hardly ever) to 4 (once a week or more). Codes for not applicable, design missing, or omitted responses were treated as missing. Primary analyses used the official index; robustness analyses substituted the complete-case item mean and each item individually. The measurement audit used 7,236 students with all three item responses. We estimated unweighted and WT2019-weighted Cronbach’s alpha, ordinal alpha from the weighted polychoric correlation matrix, corrected item-total correlations, and weighted response distributions. For regression, the official index was standardized using its WT2019-weighted mean and population standard deviation. WT2019 is the final student sampling weight provided in the SSES 2019 public-use data.

#### 2.3.2 Social–emotional skills

SSES classifies 15 skills into five Big Five–related domains (Kankaraš and Suárez-Álvarez, 2019): task performance (responsibility, self-control, persistence), emotional regulation (stress resistance, emotional control, optimism), engaging with others (energy, assertiveness, sociability), open-mindedness (curiosity, creativity, tolerance), and collaboration (empathy, co-operation, trust). We used OECD-adjusted weighted-likelihood estimates for student and parent reports and corresponding teacher indirect-assessment means (OECD, 2021b). The 15 component skills were defined within the OECD SSES assessment framework, which provides the conceptual basis for organizing them into five broader domains (Kankaraš and Suárez-Álvarez, 2019). The SSES Technical Report documents psychometric development and evaluation of the individual skill scales, including reliability and factor-analytic evidence for the student and parent assessments and reliability information for the teacher indirect assessments (OECD, 2021b). The SSES 2019 public-use files did not provide participant-level scores for the five broad OECD domains. Instead, the released scoring variables were at the skill level: adjusted weighted-likelihood estimates for the 15 core skills in the student and parent files and corresponding skill-level means in the teacher indirect-assessment file (OECD, 2021b,c). The five domain variables used here were therefore researcher-derived, framework-aligned summaries of the OECD-classified component skills rather than OECD-calibrated participant-level domain scores, and they were not treated as newly validated latent five-factor measures. Their purpose was to provide parsimonious summaries of the three conceptually related skills assigned to each OECD domain. Within each source, we standardized the three skills assigned to a domain in the Suzhou sample and averaged them equally; all three were required. We used equal weighting because the OECD framework specifies which three skills belong to each broad domain but does not provide participant-level domain scores or prescribe aggregation weights; an equal-weight mean therefore provided a transparent, reporter-comparable summary without estimating sample-specific weights. This aggregation was used for interpretive parsimony rather than to imply that the three component skills constituted a newly established unidimensional scale. Accordingly, all 15 individual skills were also analyzed separately to assess whether the domain-level pattern was broadly distributed across the component skills. The primary coefficient is therefore the change in this three-skill composite per one WT2019-weighted standard deviation of the relationship index. Sensitivity analyses (i) restandardized the completed domain composites with WT2019 and (ii) used WT2019 standardization at both skill and final-domain stages. For matched-source comparisons and descriptive correlations, each source-specific domain was WT2019-standardized within the relevant matched sample. Individual skills were also WT2019-standardized for the 45 skill-level models. The main text summarizes the domain–skill structure and reporter-specific composite construction, with corresponding public-use variable mappings provided in the Table 1; Supplementary Table S18.

**Table 1:** OECD SSES domain–skill classification and reporter-specific domain composites used in this study.

OECD domain	Component skills in the OECD classification	Student-report composite	Parent-report composite	Teacher-report composite
Task performance	Responsibility; self-control; persistence	Equal-weight mean of the three standardized student skill scores	Equal-weight mean of the three standardized parent skill scores	Equal-weight mean of the three standardized teacher indirect-assessment skill scores
Emotional regulation	Stress resistance; emotional control; optimism	Equal-weight mean of the three standardized student skill scores	Equal-weight mean of the three standardized parent skill scores	Equal-weight mean of the three standardized teacher indirect-assessment skill scores
Engaging with others	Energy; assertiveness; sociability	Equal-weight mean of the three standardized student skill scores	Equal-weight mean of the three standardized parent skill scores	Equal-weight mean of the three standardized teacher indirect-assessment skill scores
Open-mindedness	Curiosity; creativity; tolerance	Equal-weight mean of the three standardized student skill scores	Equal-weight mean of the three standardized parent skill scores	Equal-weight mean of the three standardized teacher indirect-assessment skill scores
Collaboration	Empathy; co-operation; trust	Equal-weight mean of the three standardized student skill scores	Equal-weight mean of the three standardized parent skill scores	Equal-weight mean of the three standardized teacher indirect-assessment skill scores

Domain membership follows the OECD SSES assessment framework (Kankaraš and Suárez-Álvarez, 2019; OECD, 2021a). The SSES 2019 public-use files did not include participant-level scores for the five broad domains; the authors therefore constructed the domain composites used in this study from the released skill-level estimates. OECD specifies the membership of the three skills within each broad domain but does not prescribe aggregation weights. Within each reporting source, the three standardized component skills were averaged with equal weight, with all three components required. The resulting variables are framework-aligned researcher-derived summaries rather than OECD-calibrated latent domain scores. For matched cross-informant comparisons, completed source-specific composites were subsequently WT2019-standardized within the relevant matched sample; this standardization does not establish measurement equivalence across reporting sources.

#### 2.3.3 Covariates and contextual predictors

Primary association models adjusted for the OECD socioeconomic-status index (SES), sex, and school-by-cohort fixed effects. A separate cohort main effect was not entered because it is exactly absorbed by the CohortID × SchID fixed effects. SES and the relationship index were WT2019-standardized. Relationship interactions with SES, cohort, and sex were examined separately as exploratory moderation families. Immigrant background was added only in the sensitivity analysis. The secondary prediction baseline comprised SES, cohort, sex, immigrant background, teacher-reported school climate, and principal-reported indices of school promotion of social–emotional skills, student disruptive behavior, and teacher problematic behavior. The full model added the relationship index to this identical baseline.

### 2.4 Statistical analysis

#### 2.4.1 Survey-weighted associations and source comparisons

To address RQ1, we fitted weighted least-squares models for each of five domains and three informants. Models used the SSES 2019 final student sampling weight (WT2019), adjusted for standardized SES and sex, included school-by-cohort fixed effects, and used school-by-cohort-clustered standard errors with a finite-sample correction. The coefficient is a within-school-by-cohort-unit conditional association: it compares students in the same public grouping unit after adjustment for observed covariates. The 15 relationship tests formed one Benjamini–Hochberg false-discovery-rate (FDR) family (Benjamini and Hochberg, 1995). The released WT2019 totals were 100,857.27 for age 10 and 50,106.76 for age 15, so the older cohort contributed 33.2% of the pooled weighted target despite representing 49.9% of records. We therefore repeated the domain and matched-source models after rescaling weights so that both cohorts contributed equal totals. As a design sensitivity, we also calculated jackknife repeated-replication standard errors using the 75 replicate-weight variables available in the public file and the variance rule specified in the technical report (OECD, 2021b).

To address RQ2, we formed a matched sample separately for each domain, standardized the three source-specific outcomes with WT2019 within that sample, and stacked the outcomes into long form. The weighted model included informant indicators, relationship-by-informant interactions, SES, sex, and school-by-cohort fixed effects; uncertainty was clustered by school-by-cohort unit. Linear combinations yielded each informant-specific slope and the three pairwise slope differences. The 15 contrasts formed one FDR family. Standardizing the source-specific outcomes places the coefficients on a common standard-deviation metric, but it does not establish measurement equivalence across student, parent, and teacher reports. The resulting contrasts are therefore interpreted as differences in observed standardized associations across reporting sources, not as evidence that equivalent latent constructs differ in their relationships with the predictor. Differences in these slopes may reflect reporter perspective and context, but may also reflect common-method covariance, reliability, item-form, or observability differences across reporting sources. For descriptive context, we calculated WT2019-weighted zero-order correlations within the same domain-specific matched samples: correlations among the three informant outcomes and correlations of each outcome with the student-reported relationship measure. Ninety-five percent confidence intervals for these descriptive correlations were estimated using the 75 released jackknife replicate weights. Correlations were transformed to the Fisher-*z* scale, replicate-weight variance was calculated using the OECD jackknife repeated-replication variance factor, and interval limits were then back-transformed to the correlation scale (OECD, 2021b). These correlations were not used for hypothesis testing or causal interpretation.

#### 2.4.2 Breadth, moderation, and functional form

Skill-level breadth was evaluated by applying the primary specification to all 15 standardized skills for each informant; the 45 coefficients formed one FDR family. Relationship interactions with SES, cohort, and sex were fitted as three separate exploratory families, each containing 15 FDR-adjusted tests. Cohort-stratified models provided an additional check for directional stability. Functional form was examined by adding quadratic and cubic relationship terms and jointly testing them with a two-degree-of-freedom cluster-robust Wald test; these 15 tests formed one FDR family. These analyses were exploratory and were not interpreted as subgroup effects, thresholds, or dose–response functions.

#### 2.4.3 School-by-cohort-grouped prediction

Prediction was secondary to the multi-informant association analysis. Five-fold GroupKFold cross-validation was defined by CohortID × SchID, ensuring that students from a public school-by-cohort unit were never split between training and test data in the same fold (Roberts et al., 2017). This design reduces within-unit leakage but does not verify the holdout of the same physical school across cohorts. Baseline and full models used identical folds. Preprocessing was learned from training data only; missing predictors were median-imputed within each fold, whereas outcomes were not imputed (Cawley and Talbot, 2010). The focal algorithms were Elastic Net (Zou and Hastie, 2005) and LightGBM (Ke et al., 2017), using fixed, outcome-invariant settings documented in Supplementary Methods. Models used WT2019 as sample weights. We combined held-out predictions across folds and calculated weighted *R*^2^, root mean square error, and mean absolute error. Incremental prediction was Δ*R*^2^ between otherwise identical baseline and relationship-added models. We resampled the 151 grouping units 2,000 times and recomputed Δ*R*^2^ from the fixed out-of-fold predictions; the resulting percentiles are termed *conditional grouping-unit-composition intervals* because they do not include algorithm- or tuning-selection uncertainty. Split sensitivity used five repeated shuffled grouped partitions with frozen settings. Accordingly, this procedure provides internal out-of-sample validation across held-out school-by-cohort units within the Suzhou sample; it does not constitute external validation, nor does it verify generalization to entirely unseen physical schools, cities, or education systems.

#### 2.4.4 Out-of-fold model attribution

For LightGBM, preprocessing and model fitting occurred within each training fold, after which TreeSHAP values were calculated only for the corresponding held-out units (Lundberg et al., 2020). Held-out values were concatenated to obtain one out-of-fold attribution per student. Weighted mean absolute SHAP values summarized feature ranking, and the relationship score was summarized in eight empirical bins. Because absolute prediction for adult-reported outcomes was low or sometimes negative, adult-report SHAP patterns were retained for transparent comparison but were not used to make substantive feature-rank claims. SHAP values describe a fitted predictive model; they do not identify causal importance.

### 2.5 Missing data and sensitivity analyses

Association models used outcome-specific complete cases because missingness in the focal variable was low. Matched and non-matched students were compared on SES, relationship score, cohort, and sex using weighted means or proportions and standardized mean differences. Prediction retained cases with an observed outcome and imputed predictors within training folds. Sensitivity analyses examined alternative outcome scaling, immigrant-background adjustment, alternative relationship indicators, the 75 released replicate weights, survey-weighted scaling within Elastic Net folds, cohort-stratified models, equal-cohort-total weighting, and repeated grouped validation. All retained the same non-causal interpretation. Analyses were not preregistered; the multi-informant questions were primary, whereas moderation, non-linearity, prediction, and attribution were exploratory or secondary. Analyses used Python 3.12 with pandas, NumPy, SciPy, statsmodels, scikit-learn, LightGBM, and SHAP. Construct-to-field mappings are reported in Supplementary Table S18; software versions and frozen numerical outputs are retained in the reproducibility files.

## 3 Results

### 3.1 Sample coverage and measurement

The analytical frame comprised 7,246 students in 151 school-by-cohort units identifiable in the public-use data: 3,633 students in 76 age-10 units and 3,613 in 75 age-15 units (Table 2; Figure 1). Parent records were linked for 7,080 students and teacher ratings for 7,032. Of the 7,246 students, 3,409 were female students (47.0% unweighted; 46.3% WT2019-weighted), and the OECD socioeconomic-status index had a weighted mean of 0.302 [SD = 0.846; median = 0.290, IQR (−0.370, 0.958)]. Among the 7,054 linked parent respondents with non-missing respondent identity, 62.4% were mothers or other female guardians, 36.5% were fathers or other male guardians, and 1.0% selected “other” (WT2019_PA-weighted). Of the 3,645 unique teachers who provided an indirect assessment, 3,624 (99.4%) had linked contextual-questionnaire data; among the 3,622 with non-missing sex, 73.7% were female teachers and 26.3% were male teachers (WT2019_TC-weighted). Among specifically identified subject areas, reading, writing, and literature was the most frequently reported (51.7%), followed by mathematics (27.1%), science (25.0%), and practical and vocational skills (24.3%); an additional 39.5% selected “other.” Because subject areas were multiple-response items, these percentages are not mutually exclusive (Supplementary Table S21). The relationship index was available for 7,229 students (99.77%); its unweighted mean was 50.816 (SD = 13.702), and its WT2019-weighted mean was 50.349 (SD = 14.109). Internal consistency was acceptable (weighted Cronbach’s *α* = 0.763; ordinal *α* = 0.862), and the official index correlated at 0.976 with the three-item raw mean. Complete-case samples were 7,225 for student domains, 7,054–7,056 for parent domains, and 6,929–6,948 for teacher domains. Weighted missingness was below 0.25% for the relationship index, SES, and sex and was below 0.06% for student-reported domains (Supplementary Table S14). Domain-specific matched samples included 6,784–6,804 students. Relative to excluded students, matched students were less likely to be in the age-15 cohort [standardized mean differences (SMDs) = −0.324 to −0.292] and had modestly lower SES (SMDs = −0.190 to −0.154); relationship-score and sex differences were smaller (absolute SMDs ≤ 0.075; Supplementary Table S15).

**Table 2:** Sample, linkage, and measurement characteristics.

Section	Measure	Unweighted	WT2019-weighted	Definition or note
Sample	Students	7,246	–	Analytical frame
Sample	School-by-cohort units	151	–	Public-data fixed-effect, clustering, bootstrap, and validation unit
Sample	Age-15 cohort	3,613 (49.9%)	33.2%	Age cohorts are separate cross-sections
Sample	Female	3,409 (47.0%)	46.3%	13 sex values missing
Linkage	Parent record matched	7,080 (97.7%)	–	Linked by standardized student identifier
Linkage	Teacher rating matched	7,032 (97.0%)	–	Indirect teacher assessment linked to student
Covariate	Socioeconomic status	*N* = 7,232	M = 0.302; SD = 0.846	OECD SES index
Predictor	Relationships with teachers	*N* = 7,229	M = 50.349; SD = 14.109	OECD student-perceived three-item index
Measurement	Internal consistency	Alpha = 0.752	Alpha = 0.763	Ordinal alpha = 0.862
Analysis	Matched three informant samples	*N* = 6,784–6,804	–	Domain-specific complete ratings from students, parents, and teachers

Weighted moments and proportions use WT2019. Alpha denotes Cronbach’s alpha; ordinal alpha is based on the polychoric item-correlation matrix. Outcome-specific complete-case samples are reported in the Results section.

### 3.2 Associations across reporting sources (RQ1)

More positive student-perceived relationships with teachers were associated with higher scores in all five domains for every informant. In survey-weighted models with school-by-cohort fixed effects, coefficients ranged from 0.210 to 0.245 for student outcomes, 0.075–0.089 for parent outcomes, and 0.059–0.088 for teacher outcomes. All 15 coefficients were positive and remained significant after FDR correction (maximum *q* < 0.001; Supplementary Table S1). The association was therefore not confined to student-reported outcomes, although external-informant coefficients were smaller. Standard errors based on the 75 released replicate weights averaged 0.979 times the clustered standard errors (range = 0.814–1.166) and did not change any inference (Supplementary Table S5).

### 3.3 Matched-source differences (RQ2)

Before examining the adjusted source-specific slopes, WT2019-weighted zero-order correlations in the same domain-specific matched samples provided descriptive context (Figure 2). Agreement among source-specific domain scores was positive but modest: student–parent correlations ranged from 0.168 to 0.325, student–teacher correlations from 0.096 to 0.195, and parent–teacher correlations from 0.071 to 0.193. Correlations between the student-reported relationship measure and outcomes ranged from 0.254 to 0.290 for student reports, 0.104–0.135 for parent reports, and 0.099–0.140 for teacher reports. Corresponding 95% confidence intervals, reported in Supplementary Table S22, had widths ranging from 0.046 to 0.081. These descriptive correlations show both cross-informant convergence and source dependence.

**Figure 2:**
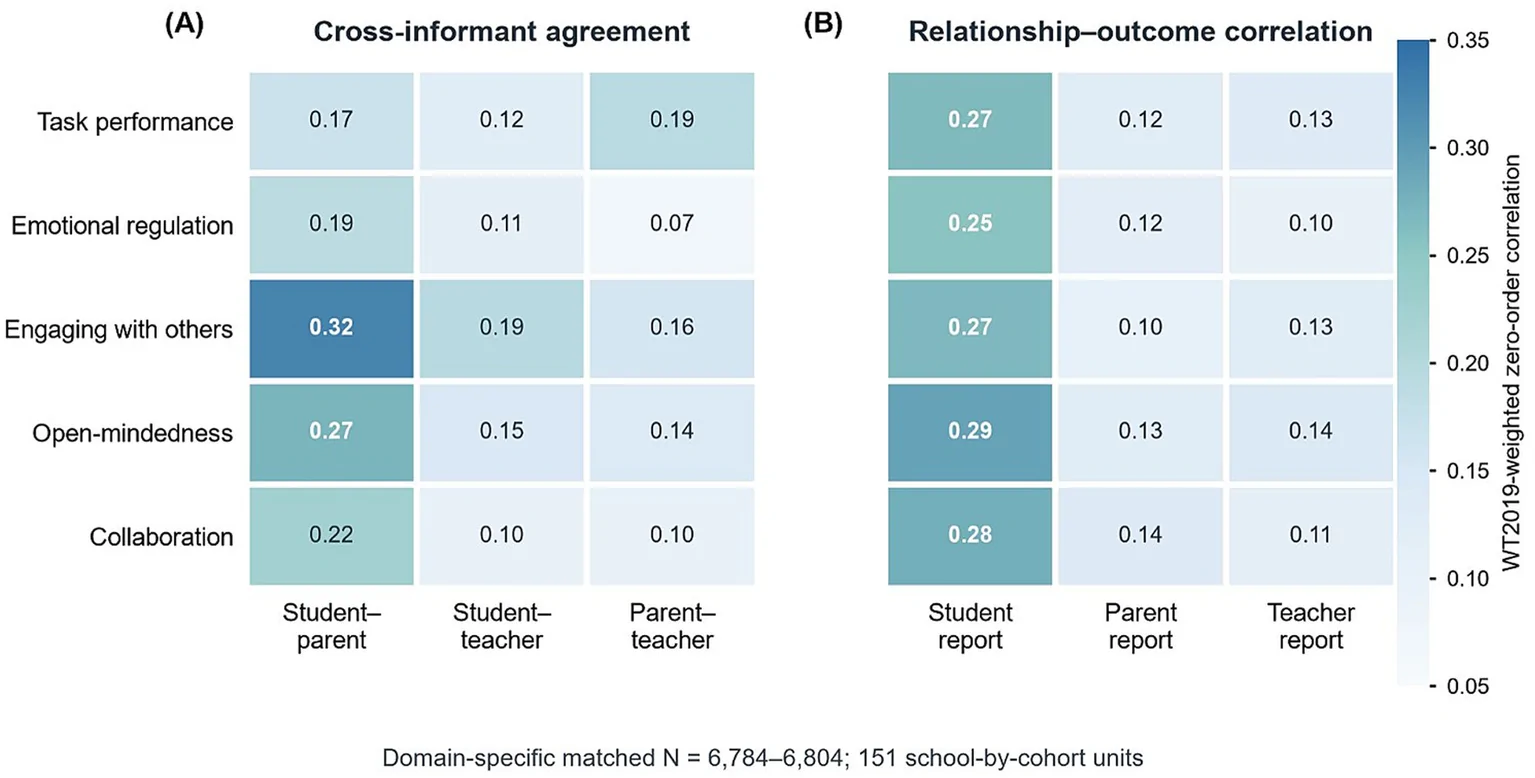
Descriptive cross-informant and relationship–outcome correlations. Cells show WT2019-weighted zero-order correlations in domain-specific samples with complete student, parent, and teacher outcomes. Corresponding 95% confidence intervals estimated using the 75 released jackknife replicate weights are reported in Supplementary Table S22. **(A)** Correlations for the three informant pairs. **(B)** Correlations between the student-reported relationship measure and each source-specific outcome. Domain-specific matched *N* = 6,784–6,804 students across 151 school-by-cohort units.

In domain-specific samples containing all three outcome sources, relationship slopes on a common WT2019-standardized scale ranged from 0.234 to 0.256 for student reports (mean = 0.244), 0.083–0.109 for parent reports (mean = 0.095), and 0.080–0.106 for teacher reports (mean = 0.096; Table 3; Figure 3; Supplementary Table S3). Parent-report slopes were smaller than student-report slopes in all five domains (differences = −0.163 to −0.136), as were teacher-report slopes (differences = −0.166 to −0.133); all 10 contrasts remained significant after FDR correction. Teacher–parent differences ranged from −0.022 to 0.023, and none remained significant after correction. Thus, the observed standardized associations differed systematically between student and adult reporting sources, whereas the parent- and teacher-report slope estimates were statistically similar.

**Table 3:** Matched-sample cross-informant associations.

Domain	Matched N	Student slope [95% CI]	Parent slope [95% CI]	Teacher slope [95% CI]	Parent–student slope difference	Teacher–student slope difference	Teacher–parent slope difference
Task performance	6,795	0.234 [0.199, 0.268]	0.093 [0.063, 0.123]	0.101 [0.074, 0.128]	−0.141 (*q* < 0.001)	−0.133 (*q* < 0.001)	0.008 (*q* = 0.678)
Emotional regulation	6,804	0.235 [0.207, 0.264]	0.100 [0.072, 0.127]	0.080 [0.055, 0.106]	−0.136 (*q* < 0.001)	−0.155 (*q* < 0.001)	−0.019 (*q* = 0.406)
Engaging with others	6,788	0.245 [0.217, 0.272]	0.083 [0.057, 0.109]	0.106 [0.079, 0.133]	−0.162 (*q* < 0.001)	−0.139 (*q* < 0.001)	0.023 (*q* = 0.328)
Open-mindedness	6,791	0.256 [0.227, 0.284]	0.092 [0.063, 0.121]	0.106 [0.079, 0.132]	−0.163 (*q* < 0.001)	−0.150 (*q* < 0.001)	0.013 (*q* = 0.546)
Collaboration	6,784	0.253 [0.224, 0.283]	0.109 [0.081, 0.138]	0.088 [0.060, 0.115]	−0.144 (*q* < 0.001)	−0.166 (*q* < 0.001)	−0.022 (*q* = 0.382)

Outcomes were WT2019-standardized within each matched domain sample. Models adjusted for sex, socioeconomic status, and school-by-cohort fixed effects; standard errors were clustered at the school-by-cohort level. Difference columns report slope contrasts and Benjamini–Hochberg-adjusted *q* values across the 15 contrasts.

**Figure 3:**
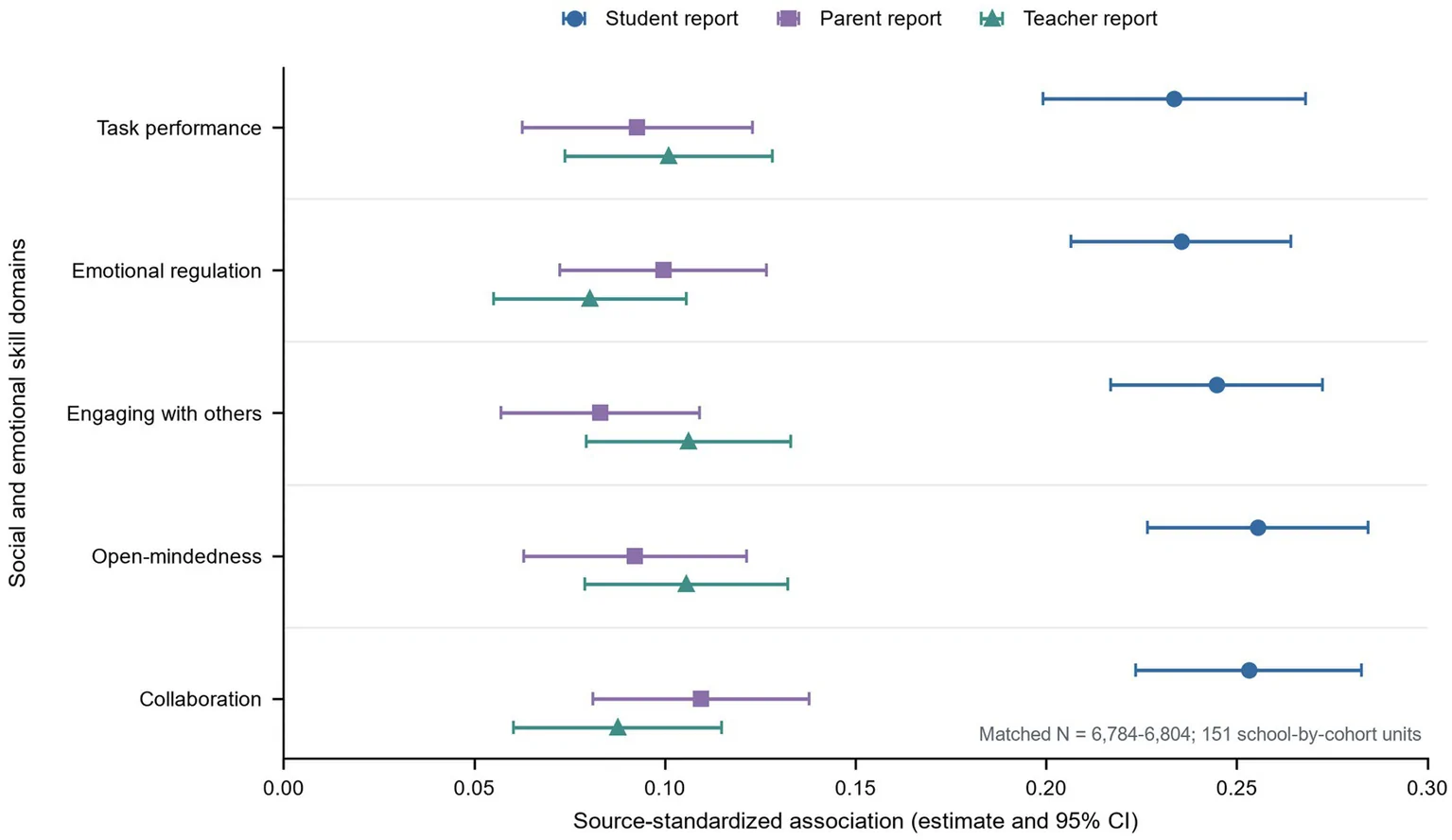
Matched-sample associations across informants. Points are source-standardized slopes, and bars are 95% school-by-cohort-clustered confidence intervals from weighted stacked models adjusted for sex, socioeconomic status, and school-by-cohort fixed effects. Domain-specific matched *N* = 6,784–6,804 students in 151 units. Numerical contrasts and false-discovery-rate-adjusted *q* values appear in Table 3.

### 3.4 Breadth and robustness

The domain pattern extended across individual skills. All 45 source-by-skill coefficients were positive, and 44 survived correction across the full family (Supplementary Table S2). Student coefficients ranged from 0.166 to 0.259 and parent coefficients from 0.061 to 0.101; all 30 survived correction. Teacher coefficients ranged from 0.023 to 0.090, and only teacher-reported stress resistance did not survive correction [*β* = 0.023, 95% CI (−0.001, 0.047), *q* = 0.059]. No relationship interaction with SES, cohort, or sex remained significant after correction in any 15-test family (Supplementary Table S6). In stratified sensitivity models, all 15 source-domain coefficients remained positive and FDR-significant at age 10 (range = 0.064–0.250) and age 15 (range = 0.039–0.228; Supplementary Table S13). Rescaling to give the two cohorts equal total weight changed a coefficient by at most 0.012 and preserved all 15 associations and the complete matched-source contrast pattern (Supplementary Tables S19–S20). Alternative outcome scaling and immigrant-background adjustment retained positive associations throughout (Supplementary Table S4), as did the raw item mean and each individual relationship item (Supplementary Table S10). Survey-weighted rather than unweighted scaling within Elastic Net folds changed Δ*R*^2^ by at most 0.0000044 (Supplementary Table S9).

### 3.5 Secondary grouped prediction

As a secondary exploratory analysis, adding the relationship index increased out-of-fold weighted *R*^2^ in all 30 source-domain-algorithm comparisons (Table 4; Figure 4; Supplementary Table S8), and 28 conditional grouping-unit-composition intervals were entirely above zero. For student outcomes, mean Δ*R*^2^ was 0.070 for Elastic Net and 0.077 for LightGBM; all 10 intervals excluded zero. Parent-report means were 0.010 and 0.013, respectively, with all intervals excluding zero. For teacher reports, Elastic Net averaged 0.008 and all five intervals excluded zero, whereas LightGBM averaged 0.010 and only three intervals did so.

**Table 4:** Summary of absolute and incremental out-of-fold prediction.

Informant	Algorithm	Mean ΔR^2^	Full-model *R*^2^ range	Intervals above zero	Repeated increments positive
Student report	Elastic Net	0.070	0.182 to 0.227	5/5	25/25
Student report	LightGBM	0.077	0.131 to 0.190	5/5	25/25
Parent report	Elastic Net	0.010	0.019 to 0.052	5/5	25/25
Parent report	LightGBM	0.013	−0.020 to 0.010	5/5	25/25
Teacher report	Elastic Net	0.008	0.051 to 0.092	5/5	25/25
Teacher report	LightGBM	0.010	0.017 to 0.053	3/5	24/25

Mean Δ*R*^2^ is averaged across the five social–emotional domains. Conditional intervals are based on 2,000 school-by-cohort-unit resamples applied to fixed out-of-fold predictions. Complete domain-level metrics are reported in Supplementary Table S8.

**Figure 4:**
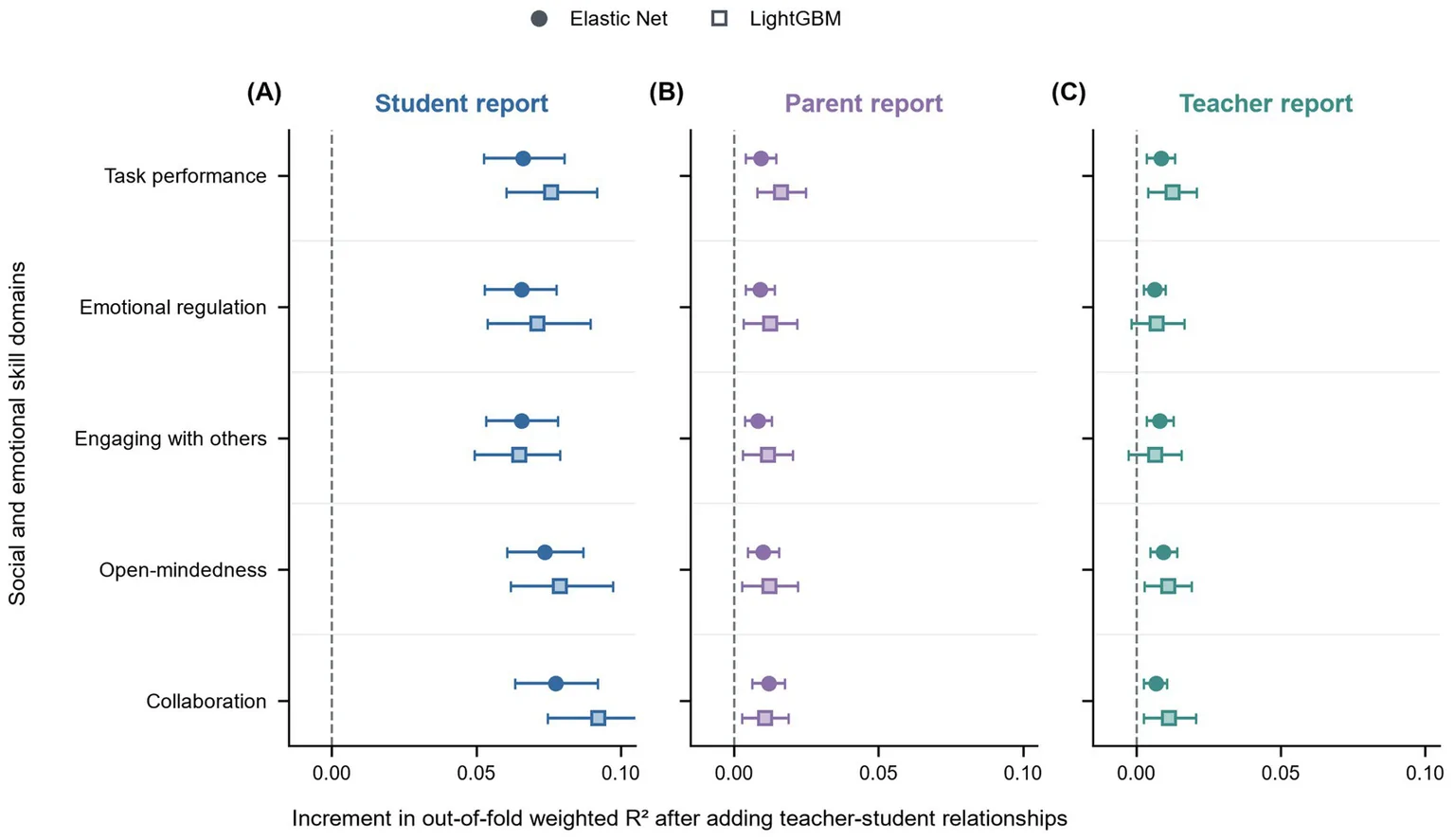
Incremental prediction in held-out school-by-cohort units. Points show the change in out-of-fold WT2019-weighted *R*^2^ after adding student-perceived relationships with teachers to the contextual baseline for **(A)** student-, **(B)** parent-, and **(C)** teacher-reported outcomes. Bars are 95% conditional grouping-unit-composition intervals from 2,000 resamples applied to fixed out-of-fold predictions. Models used five-fold GroupKFold by the public cohort-by-school identifier. Filled circles denote Elastic Net, and open squares denote LightGBM.

Absolute performance limited the interpretation. Full-model *R*^2^ ranged from 0.182 to 0.227 for student-report Elastic Net and 0.131–0.190 for student-report LightGBM. For parent outcomes, it ranged from 0.019 to 0.052 for Elastic Net but from −0.020 to 0.010 for LightGBM; teacher ranges were 0.051–0.092 and 0.017–0.053. Thus, the relationship measure added modest incremental information, but absolute predictive performance remained limited, particularly for adult-reported outcomes. Across repeated grouped partitions, all 75 Elastic Net increments and 74 of 75 LightGBM increments were positive (Supplementary Tables S16–S17).

### 3.6 Exploratory functional form and attribution

Exploratory polynomial tests rejected strict linearity in 8 of 15 source-domain models after correction: all five student domains, parent-reported engaging with others, and teacher-reported task performance and open-mindedness (Supplementary Table S7). Out-of-fold SHAP curves generally increased across the student-reported outcomes (Supplementary Figure S1; Supplementary Tables S11–S12). Adult-report curves are provided only as a reproducibility check because several corresponding LightGBM models had weak absolute performance. Taken together, positive within-unit associations were broad across informants and skills, matched slopes were systematically larger for student reports, and grouped prediction provided secondary, model-dependent triangulation.

## 4 Discussion

### 4.1 Principal findings

This study asked whether students’ perceived relationships with teachers were associated with social–emotional skills across three reporting sources and whether the magnitudes differed by source. Three findings anchor the answer. First, all 15 domain-level associations were positive and FDR-significant in within-school-by-cohort models. Second, matched student-report slopes were more than twice the adult-report slopes in every domain, whereas parent and teacher slopes did not differ reliably. Third, the pattern extended to 44 of 45 individual-skill tests. Weighted correlations independently showed modest cross-informant agreement and the same source gradient. Together, the results show a consistent reporting-source gradient in the observed associations: coefficients were largest when both the relationship measure and the social–emotional outcome were student-reported, but positive associations were also observed for parent- and teacher-reported outcomes. These comparisons do not establish that the underlying social–emotional constructs were measured equivalently across reporters.

The distinction is important. A student-reported relationship measure and student-reported skills share a survey context, response format, and evaluator; some inflation from general evaluative tendencies or common-method covariance is therefore plausible (Podsakoff et al., 2003; Primi et al., 2019). However, positive parent- and teacher-report coefficients show that the association was not entirely confined to that shared method. The similarity of the two adult slopes is also informative because parents and teachers observe students in different settings. One plausible interpretation is that the larger student-report coefficients reflect a combination of perception-linked association and shared-method covariance, whereas the smaller adult-report coefficients may reflect cross-context signal together with reporter-specific measurement and observability differences. This interpretation is consistent with recent multi-method evidence in which relationship-mediated patterns appeared for students’ self-reported social integration but not for peer nominations (Rauterkus et al., 2025).

### 4.2 Informant perspective as substantive evidence

The findings fit a multitrait-multimethod view in which observed scores reflect both the focal attribute and the method and context of observation (Campbell and Fiske, 1959). Students have privileged access to internal emotions, intentions, and generalized experiences across teachers; parents see behavior mainly in home and community settings; teachers see performance under classroom demands. Low-to-moderate agreement among informants is therefore expected and may be meaningful rather than simply erroneous (De Los Reyes et al., 2015; De Los Reyes and Epkins, 2023; Renk and Phares, 2004). The weighted correlations in Figure 2 made this architecture visible: student–parent agreement was generally higher than student–teacher or parent–teacher agreement, while relationship–outcome correlations were consistently largest for student reports.

Huang et al. (2024) provide the closest precedent for the student-report component of the present findings. Using the SSES 2019 Suzhou survey, Huang et al. reported positive associations between teacher relationships and all 15 student-reported social–emotional skills. Our student-report results recover the same broad directional pattern and therefore best serve to corroborate that earlier finding rather than establish it anew. The incremental contribution of this study is extending the analysis to matched parent- and teacher-reported outcomes and formally comparing the observed standardized association magnitudes across reporting sources. The positive, although smaller, adult-report associations indicate that the pattern is not confined to student-reported outcomes; however, as noted above, the cross-reporter contrasts may reflect both reporting perspective and reporter-specific measurement properties. SSES-based conclusions about which educational conditions matter can change when the informant changes (Guo et al., 2023), and reporting source should be prespecified rather than treated as a technical detail.

The magnitude of the present cross-informant differences can also be situated within the broader multi-informant literature. The correlations among student-, parent-, and teacher-reported domain scores in this study ranged from 0.071 to 0.325, indicating low-to-moderate correspondence. This is broadly consistent with meta-analytic evidence showing that self–other agreement for children’s and adolescents’ social competence is typically small, with stronger correspondence for some adult-informant pairs (Renk and Phares, 2004). This also aligns with more recent social–emotional skills studies reporting patterned rather than uniform agreement across students, parents, and teachers (Mudarra et al., 2022; Asadi, 2026). However, the level of agreement in the present Suzhou sample was generally lower than that reported by Guo et al. (2023) in the Finnish SSES sample, where student–parent agreement across social–emotional skills averaged approximately *r* = 0.41. Differences in cultural context, measurement form, skill aggregation, and sample composition may contribute to this variation, so the numerical magnitudes should not be treated as directly interchangeable across studies. The reporter gradient in the relationship associations was more pronounced than the cross-informant agreement pattern: matched student-report slopes were approximately 0.23–0.26, whereas parent- and teacher-report slopes were approximately 0.08–0.11. Thus, the student-report associations were roughly two to three times as large as the adult-report associations. This finding extends prior discrepancy research by showing that reporting source is related not only to agreement in social–emotional ratings but also to the magnitude of an association with a common student-perceived relational predictor. At the same time, this pattern should not be interpreted as evidence that student reports are generally more strongly related to external criteria. For example, Asadi (2026) found that teacher-reported social–emotional skills were more strongly associated with academic achievement than student- or parent-reported skills. The direction of an informant advantage therefore appears to depend on the predictor, criterion, observational context, and measurement method. In the present study, the particularly large student-report associations are consistent with both the substantive salience of students’ own relational perceptions and the possibility of shared-method covariance.

Theoretically, the most informative feature of the present pattern is not simply that the association was broad, but that its magnitude changed systematically with reporting source. The larger student-report associations are consistent with the idea that perceived teacher relationships are partly embedded in students’ own experiential and evaluative systems: the same student who experiences greater fairness, rapport, or care may also perceive their own regulation, persistence, and interpersonal functioning more positively. At the same time, the persistence of smaller positive associations in parent and teacher reports suggests that the relationship signal is not entirely confined to a shared student-report frame. This pattern is compatible with a contextual multi-informant account in which observed associations reflect both a cross-context component and reporter-specific perspective or method variance. Importantly, because measurement equivalence across reporters was not established, the present data cannot determine how much of the source gradient reflects substantive perspective *versus* measurement properties. The theoretical contribution is therefore to show that reporting source is part of the phenomenon being modeled, rather than a nuisance feature that can be removed simply by standardization.

### 4.3 Breadth across domains and skills

The association spanned task performance, emotional regulation, engaging with others, open-mindedness, and collaboration. This breadth is compatible with, although it does not test, the complementary mechanisms outlined in the Introduction. Attachment-informed accounts suggest that perceived support and responsiveness may contribute to emotional security, whereas self-determination theory emphasizes relatedness, engagement, and self-regulation; the Prosocial Classroom model further highlights supportive relationships and classroom interactions as contexts in which social–emotional behavior can be modeled and practiced (Jennings and Greenberg, 2009; Niemiec and Ryan, 2009; Verschueren and Koomen, 2012). Developmental-systems theory also cautions that these processes may be reciprocal, because students’ existing regulation and interpersonal skills can themselves shape teacher responses and students’ perceptions of those relationships (Sabol and Pianta, 2012). Meta-analyses linking relationship quality with engagement, achievement, behavior, competence, and wellbeing support this transactional interpretation (Emslander et al., 2025; Magro et al., 2024; Roorda et al., 2011, 2017). Longitudinal patterns of social–emotional development and teacher–student relationship quality further suggest heterogeneous pathways over time (Pastore et al., 2025), while reciprocal evidence cautions against privileging one direction (Magro et al., 2026; Yang et al., 2023). Because these mechanisms are broad rather than domain-specific, the cross-domain and skill-level analyses tested the breadth of the relational pattern rather than a theory-derived ordering of which social–emotional domains should show the largest associations.

The near-complete skill-level pattern reduces concern that averaging created an apparently general association from a few dominant indicators. It does not establish equal effects or validate the five-domain structure. The domain variables were researcher-constructed summaries based on the OECD classification, not official OECD domain indices. Network and validation studies suggest that the empirical organization of social–emotional skills can vary across sites and cultures (Cheung and Ng, 2024; Huo and Ning, 2025; Lee and Junus, 2024; Wang and Ning, 2026). The present domain analysis should therefore be read as an interpretable summary whose breadth was checked at the itemized skill level, rather than as a new psychometric validation. Accordingly, the domain-level results are best understood as associations with researcher-constructed summaries of OECD-designated skill groupings rather than as estimates for independently validated latent domain scores. The convergence of the skill-level analyses supports the breadth of the association pattern, but it should not be interpreted as evidence of domain-level construct validity or cross-reporter measurement invariance.

### 4.4 What grouped prediction adds—and does not add

The grouped prediction analysis addressed whether the relationship measure retained information for students in public school-by-cohort units excluded from fitting (Figure 4). Δ*R*^2^ was positive in all 30 primary comparisons and in all but one of 150 repeated algorithm-by-source-by-domain comparisons. This convergence with the weighted regressions strengthens the descriptive conclusion because the two approaches optimize different targets (Shmueli, 2010; Yarkoni and Westfall, 2017). Holding out entire public grouping units also reduced leakage that would arise if students from the same context appeared in both training and test sets (Roberts et al., 2017).

Nevertheless, the increments must be read alongside absolute performance. Prediction was strongest for student outcomes, modest for teacher outcomes, and weak for parent outcomes; several adult-report LightGBM *R*^2^ values were near or below zero. Elastic Net yielded more consistently supported increments than LightGBM for external reports, suggesting that the transferable signal was largely additive under the current feature set. Flexible algorithms do not automatically improve educational prediction, especially when outcome reliability, cross-context observability, and sample size constrain the available signal (Ke et al., 2017; Zou and Hastie, 2005). Accordingly, the prediction analysis is interpreted only as secondary evidence about incremental out-of-sample information within the present sample, rather than as an evaluation of an applied decision-making tool. Out-of-fold SHAP curves for student outcomes were broadly increasing, in agreement with the regression and ablation results (Supplementary Figure S1). Adult-report curves are provided only as a reproducibility check because several corresponding LightGBM models had weak absolute performance. SHAP and polynomial analyses describe fitted-model attribution and functional form, not mechanisms or thresholds (Lundberg et al., 2020).

### 4.5 Robustness and limitations

Several checks support the central result. The direction and inference survived alternative scaling, immigrant-background adjustment, item-level predictor substitutions, released replicate-weight variance estimation, cohort stratification, equal-cohort weighting, and repeated grouped partitions. No SES, cohort, or sex interaction survived correction; this provides no robust subgroup evidence but does not prove exact equality. The cross-sectional design remains the principal limitation. It cannot distinguish relational influence from reverse selection, stable dispositions, or unmeasured within-unit family and classroom processes. Fixed effects remove factors constant within the public grouping unit but do not supply temporal ordering or eliminate within-unit confounding. The relationship index reflects brief student perceptions of teachers collectively, whereas teacher-rated outcomes may reflect ratings from a single teacher. Reviews show that fairness, closeness, conflict, support, and autonomy are unevenly represented across relationship instruments (Phillippo et al., 2017). Source contrasts may consequently reflect reliability and item-form differences as well as perspective. More fundamentally, within-sample standardization does not establish configural, metric, or scalar equivalence across student, parent, and teacher reports. Because the reporter-specific outcomes differ in measurement form and observational context, the present slope contrasts cannot fully separate reporting perspective from differences in measurement properties.

Complete adult ratings reduced the matched samples and disproportionately excluded older and, to a lesser extent, higher-SES students. Results from those comparisons apply most directly to students with all three ratings. Public identifiers also do not establish whether repeated school codes across cohorts denote the same physical schools, so grouped validation evaluates held-out school-by-cohort units within the present Suzhou sample rather than verified unseen physical schools or external settings. The prediction protocol was neither preregistered nor nested for tuning, and its intervals condition on fixed predictions. Culturally and institutionally shaped expectations about teacher–student relationships, together with Suzhou’s specific emphasis on social–emotional education, may influence both relationship perceptions and social–emotional ratings (Chen et al., 2019; OECD, 2021c). Finally, the data represent one Chinese city in 2019; neither grouped validation nor statistical robustness establishes contemporary or cross-city transportability. The broader contribution is therefore methodological rather than numerical: the source-dependence problem applies whenever school-based decisions integrate reports from students, caregivers, and educators, whereas the coefficient magnitudes require replication in other educational and cultural systems.

### 4.6 Implications for educational psychology and practice

Students’ perceptions of fairness, rapport, and teacher care can provide contextual information when educators and educational psychologists interpret social–emotional assessment. These ratings should be interpreted as contextual components of multi-informant assessment and should not be used to infer that a higher relationship score would produce a proportional increase in social–emotional skills. Instead, practitioners can examine whether a concern appears across settings, is concentrated in the student’s experience, or differs between home and classroom reports, and then use that pattern to guide consultation with the student, family, and teachers. This approach is consistent with school-mental-health work that treats professional and family perspectives as complementary inputs rather than scores to be averaged automatically (Garner et al., 2024). Evidence linking adolescents’ perceptions of teacher–student relationships with mindfulness and distress also underscores the relevance of relational context, while its cross-sectional nature reinforces the need for cautious interpretation (Mills-Webb and Hennessey, 2025).

At the systems level, relational information may complement universal social–emotional and multi-tiered supports by identifying aspects of school experience that warrant further inquiry (Guest et al., 2024). Relationship-focused initiatives should nevertheless use prespecified outcomes, culturally responsive consultation, independent assessment where feasible, and designs capable of estimating change (Garner et al., 2024; Hoogendijk et al., 2020; Jenkins et al., 2018). The present results support multi-informant assessment practice, not selection of a specific intervention. A stronger causal test would require intervention studies that deliberately target teacher–student relationship quality and evaluate subsequent changes in students’ social–emotional skills. Where feasible, randomized or well-controlled classroom- or school-level designs would help determine whether changes in relationship quality precede and contribute to changes in social–emotional functioning rather than merely covarying with them. Future studies should repeatedly measure relationships and social–emotional skills with symmetric instruments across informants and independent observers. Longitudinal models that separate stable between-person differences from within-person change could clarify reciprocity, while fully specified multitrait-multimethod models could partition common, source-specific, and error variance. Predictive replication should hold out entire cities or education systems and repeat preprocessing and tuning inside nested outer resamples. Measurement comparability should be examined before slopes are compared across ages, cultures, or reporters.

## 5 Conclusion

Student-perceived relationships with teachers showed broad positive within-school-by-cohort associations with social–emotional skills in the SSES 2019 Suzhou sample. Associations were consistently larger for student reports but remained detectable in parent and teacher reports. For educational psychology, the central implication is that informant source changes the meaning and magnitude of social–emotional assessment results. Relationship ratings can contextualize multi-informant evidence, but they should not replace it or be interpreted as causal indicators.

## Data Availability

The data analyzed in this study are publicly available from the OECD Survey on Social and Emotional Skills (SSES) 2019 Database: https://www.oecd.org/en/data/datasets/SSES-Round-1-Database.html.
